# Wavelength-Flattened Directional Coupler Based Mid-Infrared Chemical Sensor Using Bragg Wavelength in Subwavelength Grating Structure

**DOI:** 10.3390/nano8110893

**Published:** 2018-11-01

**Authors:** Bowei Dong, Ting Hu, Xianshu Luo, Yuhua Chang, Xin Guo, Hong Wang, Dim-Lee Kwong, Guo-Qiang Lo, Chengkuo Lee

**Affiliations:** 1 Department of Electrical and Computer Engineering, National University of Singapore, Singapore 117576, Singapore; dongbowei@u.nus.edu (B.D.); elecyu@nus.edu.sg (Y.C.); 2 Institute of Microelectronics, Agency for Science, Technology and Research (A*STAR), Singapore 138634, Singapore; hut@ime.a-star.edu.sg (T.H.); kwongdl@ime.a-star.edu.sg (D.-L.K.); logq@ime.a-star.edu.sg (G.-Q.L.); 3 Center for Intelligent Sensors and MEMS (CISM), National University of Singapore, Singapore 117576, Singapore; 4 Graduate School for Integrative Science and Engineering, National University of Singapore, Singapore 117456, Singapore; 5 School of Electrical & Electronic Engineering, Nanyang Technological University, Singapore 639798, Singapore; tguoxin@ntu.edu.sg (X.G.); ewanghong@ntu.edu.sg (H.W.)

**Keywords:** nanophotonics, photonics sensors, mid-infrared, waveguide coupler, subwavelength grating

## Abstract

In this paper, we report a compact wavelength-flattened directional coupler (WFDC) based chemical sensor featuring an incorporated subwavelength grating (SWG) structure for the mid-infrared (MIR). By incorporating a SWG structure into directional coupler (DC), the dispersion in DC can be engineered to allow broadband operation which is advantageous to extract spectroscopic information for MIR sensing analysis. Meanwhile, the Bragg reflection introduced by the SWG structure produces a sharp trough at the Bragg wavelength. This sharp trough is sensitive to the surrounding refractive index (RI) change caused by the existence of analytes. Therefore, high sensitivity can be achieved in a small footprint. Around fivefold enhancement in the operation bandwidth compared to conventional DC is achieved for 100% coupling efficiency in a 40 µm long WFDC experimentally. Detection of dichloromethane (CH_2_Cl_2_) in ethanol (C_2_H_5_OH) is investigated in a SWG-based WFDC sensor 136.8 µm long. Sensing performance is studied by 3D finite-difference time domain (FDTD) simulation while sensitivity is derived by computation. Both RI sensing and absorption sensing are examined. RI sensing reveals a sensitivity of −0.47% self-normalized transmitted power change per percentage of CH_2_Cl_2_ concentration while 0.12% change in the normalized total integrated output power is realized in the absorption sensing. As the first demonstration of the DC based sensor in the MIR, our device has the potential for tertiary mixture sensing by utilizing both changes in the real and imaginary part of RI. It can also be used as a broadband building block for MIR application such as spectroscopic sensing system.

## 1. Introduction

To implement industrial process control, security and surveillance, environmental analysis, and clinical/biomedical monitoring, numerous sensors with small footprints, high stability, low cost, and low power consumption are demanded. Nanophotonics sensors are promising to fulfill these goals [1,2,3,4,5,6]. There are two mainstream configurations for nanophotonics sensors, namely out-of-plane configuration and in-plane configuration [7,8,9,10,11,12,13,14]. In out-of-plane configuration, light is routed vertically to the sensing area and interacts with the analytes. The reflected or transmitted light is received for analysis. While nano-aperture optical tweezers are used to trap and localize particles [15], plasmonic structures are employed to concentrate the electromagnetic (EM) wave in order to enhance light-matter interaction [16,17,18,19,20,21]. Up to date, blood analysis, serum analysis, genome detection, biological toxin detection, beer analysis and carbon dioxide sensing have been well explored and realized using out-of-plane configuration [22,23,24,25,26,27]. In in-plane configuration, light is directed to waveguides either by grating coupler, tapered waveguide coupler or integrated laser. Then the light travels through the waveguide and reaches the sensing area to interact with the analytes; and finally it is routed to the detector through output waveguides [28,29,30]. Compared to out-of-plane configuration, in-plane configuration offers advantages such as on-chip integrability with light sources and detectors that can lead to better scalability, and larger optical path-to-cell volume ratio which will guarantee more compact sensors while maintaining high sensitivity [31,32,33]. Besides many demonstrations of different types of sensors relying on in-plane configuration, such as spiral waveguide sensors [34,35,36], ring/disk resonator sensors [37,38], slot waveguide sensors [39,40,41], and plasmonics based sensors [42,43], directional coupler (DC) as a key component for light routing and power splitting has also been explored as a nanophotonics sensor [44,45]. However, on the one hand, the operation bandwidth of these DC sensors is limited due to dispersion; on the other hand, a large footprint is required for the sensors to interact with the analyte in order to produce significant phase difference. Furthermore, to the best of our knowledge, DC sensor has not been reported in the mid-infrared (MIR) wavelength region. MIR is an important wavelength range in the EM wave spectrum since it contains the fingerprints of many chemicals and biological agents [46,47,48,49,50,51]. Not only will MIR enable label-free and damage-free sensing [52], but also MIR sensing can utilize both changes in the real part and imaginary part of RI to allow tertiary mixture sensing [53].

Subwavelength grating (SWG) structure, in which the period of the grating is much smaller than the operating wavelength, can effectively suppress diffraction. As a result, the structure behaves like a homogeneous material for the incoming EM wave. The properties of SWG structure can be adjusted effectively by properly designing its period and duty cycle. Thanks to the advances in nanofabrication technology, SWG structure can be fabricated with a precisely controlled small period for mid or near infrared applications [54]. SWG has been deployed in photonics systems for both telecommunication applications [55,56,57] and sensing applications [58,59]. Incorporating SWG structures into DC sensors can bring about two advantages. Firstly, the dispersion in DC can be engineered to achieve broadband operation that benefits spectroscopic sensing. Secondly, the Bragg wavelength introduced by the SWG structure is sensitive to the refractive index (RI) change of the surroundings. Hence, by leveraging the Bragg wavelength-induced sensitivity enhancement, a more compact sensor can be realized.

In this paper, we report a compact wavelength-flattened DC (WFDC, or broadband DC) based sensor for the MIR. A SWG structure which engineers dispersion to allow broadband operation is incorporated in DC while it also introduces a sharp trough at the Bragg wavelength for high sensitivity. Around fivefold enhancement in the operation bandwidth compared to conventional DC has been achieved for 100% coupling efficiency in a 40 µm long SWG-based WFDC. The sensing capability for dichloromethane (CH_2_Cl_2_) detection in ethanol (C_2_H_5_OH) is investigated by simulation in a device of 136.8 µm long. A sensitivity of −0.47% change in the self-normalized transmitted power per percentage of CH_2_Cl_2_ concentration is revealed in the RI sensing while 0.12% change in total integrated output power is realized in absorption sensing. As the first investigation of a DC based MIR sensor, our device can utilize both the absorption and RI change caused by analytes to potentially allow tertiary mixtures sensing. It can also be adopted as a promising broadband component for light routing and power splitting in MIR spectroscopic sensing systems.

## 2. Concept and Design Optimization

**Figure 1:**
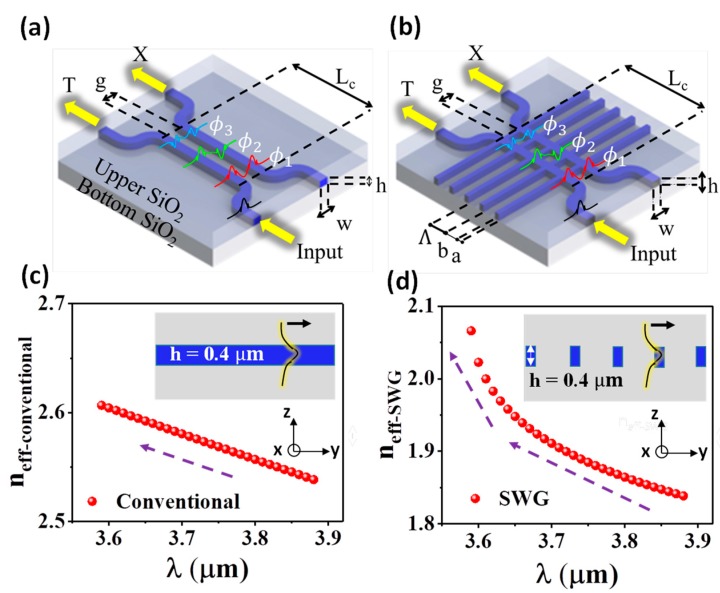
(**a**) Schematic of the conventional directional coupler (DC) and (**b**) the SWG-based wavelength-flattened directional coupler (WFDC) in SOI platform. T is the transmitted power through the input waveguide. X is the evanescently coupled power to the adjacent waveguide. w, h, g and $L_{c}$ are the waveguide width, waveguide height, coupling gap and coupling length, respectively. Λ, a and b are the SWG period, silicon width and silicon dioxide width respectively. *ɸ*_1_, *ɸ*_2_ are the even mode and the odd mode presented in the DC. *ɸ*_3_ is a weakly coupled even mode. (**c**) Dispersion of the fundamental mode in a slab waveguide as shown in the inset. The slab waveguide has 0.4 µm thick Si guiding layer and infinitely thick silicon dioxide cladding. (**d**) Dispersion of the floquet mode in the SWG as shown in the inset. The black glowing line in the insets of (**c**,**d**) show the E-field of the EM wave as it propagates in the waveguide.

Figure 1a shows the schematic of conventional DC which consists of two slightly spaced waveguides. Owing to evanescent wave coupling, an even mode *ɸ*_1_ and an odd mode *ɸ*_2_ exist in the coupled structure according to the coupled mode theory (CMT). The input EM wave excites both *ɸ*_1_ and *ɸ*_2_. The coupling between these two modes allows the EM wave to transfer between these two waveguides. The required coupling length for 100% coupling efficiency is analytically calculated by $L_{\pi }\;=\;(\lambda /2)/(n_{eff1}\;-{\;n}_{eff2})$, where λ is the wavelength and *n_eff_*_1_ and *n_eff_*_2_ are the effective RI of modes *ɸ*_1_ and *ɸ*_2_ respectively [60]. As λ decreases, the stronger modal confinement tends to equate *n_eff_*_1_ and *n_eff_*_2_, resulting in the significant drop in *n_eff_*_1_ − *n_eff_*_2_. Hence, the desired coupling efficiency can only be achieved in a limited wavelength range.

The schematic of our MIR SWG-based WFDC is illustrated in Figure 1b. The coupling region is formed by inserting SWG into the conventional DC structure. The RI of the equivalent homogeneous material is determined by period Λ and duty cycle = a/Λ. In our study, silicon-on-insulator (SOI) is the chosen material platform since its fabrication is mature and stable.

In order to illustrate how SWG structure helps to increase the operation bandwidth of DC, we compare the dispersion of waveguide with and without SWG structure. Figure 1c shows the theoretical dispersion of the fundamental mode in a slab waveguide with $h\;=\;0.4\;\mu m,\;n_{\mathrm{si}}\;=\;3.4,\;n_{{\mathrm{SiO}}_{2}}\;=\;1.4$ and infinitely thick SiO_2_ cladding. The dispersion is simulated by finite difference analysis using Lumerical Mode Solution. Figure 1d presents the dispersion of the floquet mode in an SWG with Λ = 0.86 μm and duty cycle = 0.25 while the rest of the parameters are the same as slab waveguide. Floquet mode’s dispersion is calculated numerically by the effective medium theory which will be explained in details in the next paragraph. Unlike the linear dispersion presented in the slab mode, the effective RI of the floquet mode in SWG rises drastically as λ approaches the Bragg wavelength $\lambda_{B}$. Such tremendous effective RI boost has a different influence on *n_eff_*_1_ and *n_eff_*_2_. *n_eff_*_1_ is appreciably elevated while *n_eff_*_2_ is less affected by the index perturbation due to *ɸ*_2_’s anti-symmetry [61]. The resultant increase in *n_eff_*_1_ − *n_eff_*_2_ compensates for its reduction as λ decreases. Consequently, *n_eff_*_1_ − *n_eff_*_2_ is preserved and WFDC can be realized.

**Figure 2:**
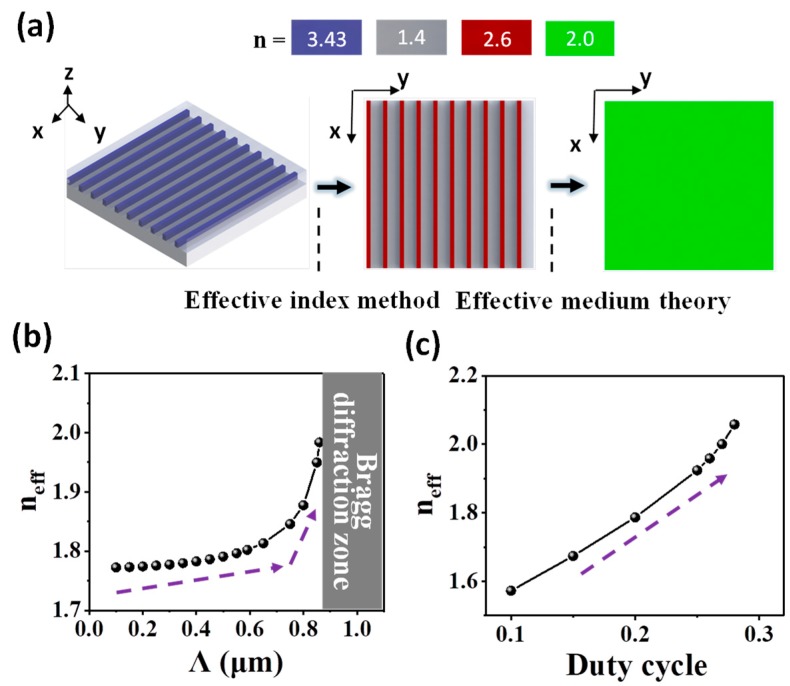
(**a**) The method to obtain the effective RI *n_eff_* of the equivalent homogeneous material of SWG. The effective index method is firstly used to compress the 3D SWG (left) to a 2D SWG (middle) by reducing the z dimension. Then the effective RI *n_eff_* of the equivalent homogeneous material (right) is derived by the effective medium theory. (**b**) The dependence of *n_eff_* on Λ at 3.62 µm when duty cycle = 0.25. (**c**) The dependence of *n_eff_* on duty cycle at 3.62 µm when Λ = 0.86 µm.

Figure 2a demonstrates our method to obtain the RI of the equivalent homogenous material of 3D SWG on the SOI platform. The study is conducted for the wavelength of 3.62 µm, assuming Λ = 0.86 µm and duty cycle = 0.25 without loss of generality. The 3D SWG structure (left) is firstly compressed into an equivalent 2D SWG by reducing the z dimension using the effective index method. Here, we use the commercial simulation tool Lumerical Mode Solution to derive the effective RI. After this step, the 3D SWG can be regarded as a 2D SWG in the xy plane (middle). The red strips possess an effective RI of 2.6 determined by the effective RI of the fundamental mode of 0.4 μm Si slab covered by SiO_2_ cladding. The grey strips have an effective RI of 1.4 since it is compressed from a structure consisting solely of SiO_2_. Then, according to Amnon Yariv and Pochi Yeh [62], the effective RI *n_eff_* of the equivalent homogenous material of the compressed 2D SWG can be analytically solved by:

(1) $$\;n_{\mathrm{eff}}\;=\;\frac{\mathrm{cK}}{\omega }\;$$

(2) $$\;{\cos K\Lambda \;=\;\cos(k}_{1x}{a)\cos(k}_{2x}b)\;-\;\Delta \sin\left(k_{1x}a\right)\sin\left(k_{2x}b\right)\;$$

(3) $$\;\Delta \;=\;\frac{1}{2}\left(\frac{k_{2x}}{k_{1x}}\;+\;\frac{k_{1x}}{k_{2x}}\right)\;$$

(4) $$\;k_{1x}\;=\;\sqrt{{\left(\frac{\omega }{c}n_{1}\right)}^{2}\;-{\;\beta }^{2}}\;$$

(5) $$\;k_{2x}\;=\;\sqrt{{\left(\frac{\omega }{c}n_{2}\right)}^{2}\;-{\;\beta }^{2}}\;$$

where c = 3 × 10^8^ m/s is the speed of light in vacuum, K is the Bloch wave number, ω is the angular frequency of the EM wave determined by the wavelength, Λ is SWG’s period while a and b equal to [Λ × duty cycle] and [Λ × (1 − duty cycle)], respectively, *n*_1_ and *n*_2_ are the effective RI of the two material layers in the 2D structure (in our case *n*_1_ = 2.6 and *n*_2_ = 1.4), k_1x_ and k_2x_ are the wave vector along the propagation direction, and β is the projection of the wave vector along the boundary plane which equals to 0 since normal incidence is assumed in our study. After numerical calculations using Equations (1)–(5), the 2D SWG in the middle is finally simplified to an equivalent homogeneous material with RI = 2.0 (right).

Since the solution is determined by Λ = a + b and duty cycle = a/Λ in the SWG structure, we optimize these two parameters accordingly. The targeted wavelength range for flattening is 3.66–3.895 µm which is available in our laser setup. We fix the duty cycle at 0.25 first in order to optimize Λ. Initially, we aim to locate the Bragg wavelength $\lambda_{B}$ only slightly below 3.66 µm whereby *n_eff_* increases most significantly to compensate for the drop in *n_eff_*_1_ − *n_eff_*_2_ as λ decreases. Nonetheless, this scheme is risky. In the case when Λ of the fabricated device is larger than the design due to fabrication imperfection, the Bragg wavelength will red shift and become $\lambda_{B}^{\prime }\;>\;3.66\;µm.$ Subsequently, a small wavelength range of ${(\lambda }_{B}^{\prime }\;-\;3.66)\;µm$ will undergo Bragg diffraction. To minimize this risk, we position the Bragg wavelength at 3.62 µm instead. Figure 2b presents the Λ optimization result. *n_eff_* boosts at Λ = 0.75 µm after a gradual rise from Λ = 0.1 µm to Λ = 0.75 µm. A maximum *n_eff_* = 1.984 is reached at Λ = 0.86 µm, after which, mathematically, Equation (2) is not solvable since the right hand side is larger than unity and the Bragg diffraction happens physically. Thus, 0.86 µm is chosen as the optimized Λ.

Duty cycle mainly affects the excitation of *ɸ*_3_ in the SWG-based DC (see *ɸ*_3_ in Figure 1b). *ɸ*_3_ is interpreted as the supermode caused by the coupling of the second order modes of individual waveguides. Once excited, *ɸ*_3_ could interfere with *ɸ*_1_ and *ɸ*_2_ to cause spurious power transfer. Duty cycle around 0.2 is chosen since it could effectively suppress the excitation of *ɸ*_3_ as suggested by Halir et al. [61]. Figure 2c plots the dependence of *n_eff_* on the duty cycle. A positive quasi-linear relation is observed. This positive relation is reasonable since larger duty cycle grants a higher Si ratio in the SWG to elevate the effective RI. Although a smaller duty cycle could more effectively suppress *ɸ*_3_, it requires more stringent fabrication. Subsequently, duty cycle = 0.25 is selected such that the critical dimension of 215 nm is 20% larger than our current fabrication limit of 180 nm linewidth using 248 nm DUV lithography.

## 3. Device Fabrication and Characterization

The waveguide dimension of h × w = 0.4 μm × 1.2 μm is chosen to achieve low loss single mode waveguide [29]. The gap *g* for the conventional DC and SWG-based WFDC are 0.5 µm and 1 µm respectively. The length of the grating in SWG is 10 µm. Λ = 0.86 µm and duty cycle = 0.25 are selected as the center parameters while some variations are considered to investigate the influence of Λ and duty cycle on coupling efficiency. Conventional DC and SWG-based WFDC with varying L_c_s are fabricated in order to achieve different coupling efficiencies.

**Figure 3:**
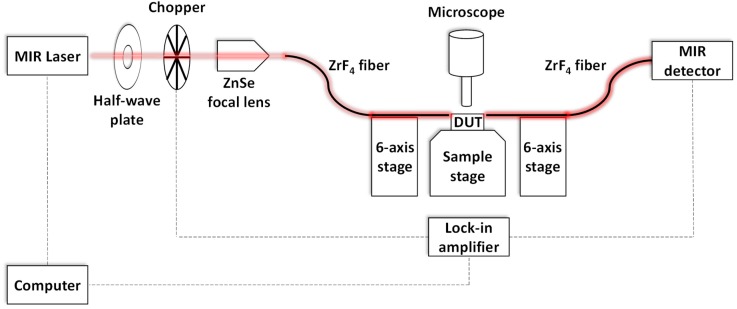
Experimental setup for optical testing. The dashed lines and glowing lines show the equipment connection and light path respectively.

The fabrication starts from a commercially available 8-inch SOI wafer with a 220 nm Si device layer and 3 µm SiO_2_ BOX. A 180 nm silicon blanket is epitaxially grown to top up the device layer to 400 nm. The devices are patterned by deep ultra-violet (DUV) photolithography followed by silicon reactive ion etching (RIE). Cladding oxide of 3 µm is then deposited by plasma enhanced chemical vapour deposition (PECVD). Finally, a deep trench with more than 100 µm in depth is etched for butt fiber coupling. The experimental setup for optical testing is presented in Figure 3. The dashed lines show the equipment connection while the glowing lines exhibit the light path. Light is emitted from the MIR laser (Daylight Solution) and passes through a half-wave plate (Thorlab) for polarization control. Transverse-electric (TE) mode is used in the experiment. Next, the light is modulated by a chopper which serves as an external reference signal to the lock-in amplifier (Stanford Research System) to reduce MIR detector noise. The light is then launched to the ZrF_4_ MIR fiber (Thorlab) and coupled to the device sitting on the sample stage (Kohzu). Fine fiber alignment is achieved by the 6-axis stage. Finally, the output light is captured by another MIR fiber and routed to the MIR detector (Horiba).

**Figure 4:**
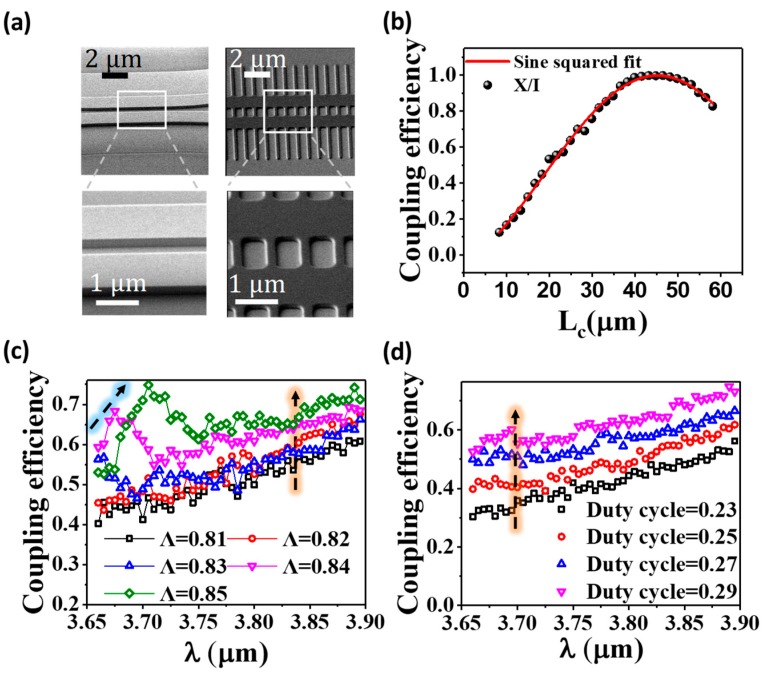
(**a**) The scanning electron microscope (SEM) images of conventional DC and SWG-based WFDC. (**b**) The DC behavior of the SWG-based WFDC with Λ = 0.83 µm and duty cycle = 0.25 at 3.7 µm. The data fits well with the sine squared function with adj. R-square of 0.997, showing good performance of the SWG-based WFDC. (**c**) The influence of Λ on coupling efficiency. Duty cycle and the number of SWG periods are fixed at 0.25 and 30 respectively. (**d**) The influence of duty cycle on coupling efficiency. Λ and the number of SWG periods are fixed at 0.81 µm and 30 respectively.

Figure 4a shows the scanning electron microscopy (SEM) images of the fabricated devices. Smooth sidewalls and sharp gaps are observed. These guarantee low loss of SWG-based WFDC. An actual Λ of 0.83 µm is realized though the nominal value is 0.86 µm. To ensure that the SWG-based WFDC exhibits good DC performance, we measured the self-normalized coupled power X/I at several L_c_, where *X* is the power coupled evanescently through the DC and I = X + T is the total power measured at the DC output (See Figure 1a). According to the theoretical DC model analyzed by the CMT, X/I should satisfy the sine squared function:

(6) $$\;\frac{X}{I}{\;=\;\sin}^{2}\left(\frac{\pi }{2}\frac{L_{c}}{L_{\pi }}\;+\;\emptyset_{0}\right)\;$$

The experimental result of X/I vs. L_c_ at 3.7 µm is shown in Figure 4b. The data is fitted well by the sine squared function with adj. R-square of 0.997, demonstrating the good DC performance of our SWG-based WFDC.

We study the influence of Λ on the coupling efficiency of our SWG-based WFDC. Λ is varied from 0.81 µm to 0.85 µm in steps of 0.01 µm while the duty cycle and the number of SWG periods are fixed at 0.25 and 30 respectively. The result is presented in Figure 4c. Devices with Λ = 0.81 µm and Λ = 0.82 µm show gradual increase of coupling efficiency throughout the wavelength range of 3.66–3.895 µm. In contrast, a local maximum of coupling efficiency can be observed in devices with Λ = 0.83 µm, 0.84 µm and 0.85 µm. Meanwhile, the local maximum shifts to a longer wavelength with increasing Λ as indicated by the blue dashed arrow in Figure 4c. These local maximums are caused by the rapid increase of *n_eff_* as the wavelength approaches the Bragg wavelength (see Figure 2b). The high *n_eff_* enhances the coupling between the waveguides, leading to higher coupling efficiency. The local maximum shifts due to the fact that larger Λ corresponds to larger Bragg wavelength according to $\lambda_{B}{\;=\;2n}_{\mathrm{eff}}\Lambda$. Apart from the local maximum, another observation is that higher Λ provides stronger coupling at individual wavelength as indicated by the orange dashed arrow. This could be attributed to the stronger coupling offered by higher *n_eff_* since the larger Λ is closer to the Bragg diffraction zone as illustrated in Figure 2b.

The dependence of coupling efficiency on the duty cycle is shown in Figure 4d. Λ and the number of SWG periods are fixed at 0.81 µm and 30 respectively. As the duty cycle varies from 0.23 to 0.29 in steps of 0.02, the increasing trend of coupling efficiency throughout 3.66–3.895 µm maintains, revealing that *ɸ*_3_ is successfully suppressed by the small duty cycle to achieve a stable coupling. The coupling efficiency is positively related to duty cycle at each individual wavelength as indicated by the orange dashed arrow. This is a result of higher duty cycle offering higher *n_eff_* (see Figure 2c) so that coupling is strengthened. Additionally, this suggests that SWG could be adopted in DC to reduce the device footprint as well due to its capability of offering stronger coupling.

**Figure 5:**
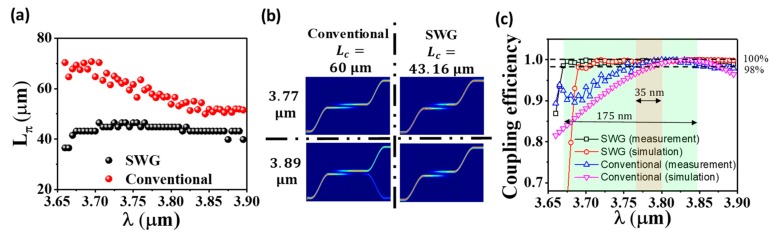
(**a**) Comparison of experimental L_π_ at different λ  in conventional DC and SWG-based WFDC. (**b**) The simulated mode profile of conventional DC and SWG-based WFDC at 3.77 µm and 3.89 µm for 100% coupler efficiency. The simulation is performed using Lumerical FDTD Solutions. (**c**) The WF performance of SWG-based WFDC compared with conventional DC for 100% coupling efficiency. The green and red area respectively highlight the operation bandwidth of SWG-based WFDC and conventional DC.

In the following discussions, the devices are all designed with the same Λ = 0.83 µm and duty cycle = 0.25. From Figure 4b, we could identify L_π_ which provides 100% coupling efficiency for 3.7 µm EM wave. Similarly, we extract L_π_ at individual wavelength for both conventional DC and SWG-based WFDC. The result is shown in Figure 5a. It is clear that L_π_ drops almost linearly in conventional DC and yet is more stable in SWG-based WFDC. We define the percentage change in L_π_ as:

(7) $$\;\frac{L_{\pi \max}\;-{\;L}_{\pi \min}}{L_{\pi \min}}\;$$

In conventional DC, the percentage change in L_π_ is 41.4% while our SWG-based WFDC could achieve 16.7%. A more stable L_π_ suggests the SWG-based WFDC is more resistant to wavelength change compared to conventional DC. Figure 5b presents the comparison of simulated mode profiles of conventional DC and SWG-based WFDC under different conditions. The simulation is performed using Lumerical FDTD Solutions [63]. For conventional DC, while L_c_ = 60 µm guarantees 100% coupling efficiency at 3.77 µm, a substantial amount of power is transmitted through the original waveguide as the wavelength rises to 3.89 µm. Thus, 100% coupling efficiency is compromised. However, in SWG-based WFDC with L_c_ = 43.16 µm, 100% power coupling ratio could be maintained even if the wavelength changes from 3.77 µm to 3.89 µm.

Figure 5c shows the wavelength-flattened performance by comparing the operation bandwidth of SWG-based WFDC with that of conventional DC at 100% coupling efficiency respectively. The acceptance range is defined as 98–100%. Similar to our previous work, the lower limit and upper limit of the 98–100% range is defined as the first wavelength that stays in this range and the wavelength with the highest coupling ratio respectively [64]. As shown in Figure 5c where L_c_ = 43.16 µm in the SWG-based WFDC, the coupling efficiency is maintained between 98% and 100% over the wavelength range of 3.67–3.845 µm (175 nm span). Nevertheless, in conventional DC with L_c_ = 49 µm, the corresponding wavelength range is only 3.765–3.8 µm (35 nm span). Fivefold enhancement is realized for 100% coupling efficiency. The drastic drop of coupling efficiency (or the trough) at 3.67 µm is observed which could be explained by the SWG reflection as wavelength approaches the Bragg wavelength. This trough is utilized for RI sensing in the following context. The simulation results of coupling efficiency derived by 3D finite-difference time domain (FDTD) simulation are also presented in Figure 5c, which is consistent with the experimental data.

## 4. Investigation of Sensing Performance

**Figure 6:**
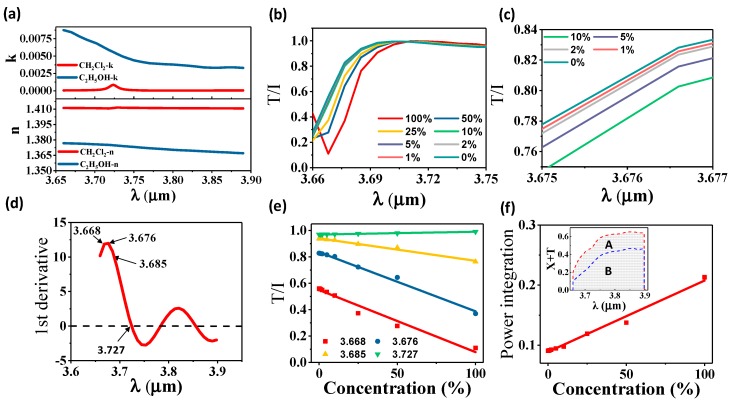
Simulated sensing results of CH_2_Cl_2_ detection in C_2_H_5_OH using 3D FDTD simulation. (**a**) Wavelength dependent RI of CH_2_Cl_2_ and C_2_H_5_OH adopted from [65]. The upper and lower panel show the imaginary part and real part respectively. (**b**) Self-normalized transmitted power (T/I) spectrum in different CH_2_Cl_2_ concentration. (**c**) Zoom-in self-normalized transmitted power (T/I) for low CH_2_Cl_2_ concentration sensing. (**d**) The first derivative derived from 0% CH_2_Cl_2_ curve in (**b**). (**e**) The self-normalized transmitted power (T/I) versus CH_2_Cl_2_ concentration at different wavelengths. The sensitivities can be extracted from the slope of the fitted linear curves. (**f**) Normalized total integrated output power (X + T) versus concentration. The slope shows the sensitivity. Inset: Spectrum of X + T. Area B is the power integration for pure C_2_H_5_OH while Area A + B is the power integration for pure CH_2_Cl_2_.

Dichloromethane (CH_2_Cl_2_) is a germinal organic liquid with important applications in industry as a solvent. The detection of CH_2_Cl_2_ is critical since it is hazardous while being colorless and volatile. Here we investigate the sensing capability of our device for CH_2_Cl_2_ detection in ethanol (C_2_H_5_OH) by simulation. Figure 6a shows the complex RI of both CH_2_Cl_2_ and C_2_H_5_OH. The imaginary part of RI of CH_2_Cl_2_ is much lower than that of C_2_H_5_OH, indicating mixture with higher CH_2_Cl_2_ concentration will cause weaker light attenuation. The difference in the real part of RI between CH_2_Cl_2_ and C_2_H_5_OH exceeds 0.03 across 3.65 µm to 3.9 µm. Such a difference is able to induce a significant shift of Bragg wavelength when CH_2_Cl_2_ concentration changes in the mixture. The complex RI of CH_2_Cl_2_ and C_2_H_5_OH are adopted from [65]. The complex RI of the mixture is calculated using Arago-Biot equations which states both the real and imaginary part of RI of the mixture are the linear combination of the two ingredients with their concentration as the linear coefficients respectively [66].

We investigate RI sensing and absorption sensing enabled by the change of the real part and imaginary part of RI, respectively, by simulation performed in a device 136.8 µm long using Lumerical FDTD Solutions. For RI sensing, the normalized transmitted power (T/I) spectrum of mixtures with different CH_2_Cl_2_ concentration is presented in Figure 6b. The trough blue shifts due to the rising surrounding effective RI caused by the drop of CH_2_Cl_2_ concentration. Figure 6c presents the zoom-in of Figure 5b to the low CH_2_Cl_2_ concentration region for better visualization. The first derivative of 0% CH_2_Cl_2_ concentration curve in Figure 6b is derived and plotted in Figure 6d. The magnitude of this first derivative indicates the sensitivity of T/I to wavelength change, and thus RI sensitivity. Four wavelengths namely 3.668 µm and 3.676 µm with high first derivative, 3.685 µm with a medium first derivative, and 3.727 µm with a near-zero derivative are studied. T/I is plotted against different CH_2_Cl_2_ concentrations in Figure 6e. The slopes of the fitted curves represent the sensitivity at each wavelength. Sensitivity of −0.47%, −0.45%, −0.17%, and 0% T/I change per percentage of CH_2_Cl_2_ concentration is demonstrated at 3.668 µm, 3.676 µm, 3.685 µm, and 3.727 µm respectively. This result shows that the first derivative of the T/I spectrum serves as a good reference for the selection of sensing wavelength as its high value returns high sensitivity while its near-zero value returns near-zero sensitivity.

The capability of absorption sensing is also examined. This sensing mechanism is especially enabled by operating in the MIR region. Since CH_2_Cl_2_ and C_2_H_5_OH have distinct imaginary parts of RI, their mixture shows different absorption strength in changing CH_2_Cl_2_ concentrations. As shown in the inset of Figure 6f, [area B (green)] shows the integration of total output power (X + T) over the spectrum when the mixture is free of CH_2_Cl_2_ while [area A (grey) + area B (green)] presents the integration when the mixture is free of C_2_H_5_OH. Pure CH_2_Cl_2_ allows stronger light transmission since its low imaginary part of RI causes less absorption. We plot the integration of X + T (or power integration) of the mixture with different CH_2_Cl_2_ concentrations. The slope of the fitted linear curve represents a sensitivity of 0.12% change in the normalized total integrated output per percentage of CH_2_Cl_2_ concentration in absorption sensing.

## 5. Conclusions

In summary, we design, fabricate, and characterize a compact wavelength-flattened directional coupler based chemical sensor for the MIR. Broadband performance is achieved by incorporating a subwavelength structure to the directional coupler for dispersion engineering. Meanwhile, the sensitive trough at the Bragg wavelength introduced by the subwavelength grating structure allows a compact sensor with high sensitivity to RI change. Around fivefold enhancement in the operation bandwidth compared to the conventional directional coupler is demonstrated experimentally for 100% coupling efficiency in the device with a small length of ~40 µm. Dichloromethane (CH_2_Cl_2_) detection in ethanol (C_2_H_5_OH) is investigated by 3D FDTD simulation to examine sensing performance and obtain sensitivity. The sensing capability of a device with 136.8 µm length reveals −0.47% change in the self-normalized transmitted power per percentage of CH_2_Cl_2_ concentration in RI sensing, while 0.12% change in total integrated output power is realized in absorption sensing. Our device can potentially work for sensing of tertiary mixture as well as for MIR applications that require broadband operation such as spectroscopic sensing systems.
